# PPAR*α*: A Master Regulator of Bilirubin Homeostasis

**DOI:** 10.1155/2014/747014

**Published:** 2014-07-23

**Authors:** Cyril Bigo, Jenny Kaeding, Diala El Husseini, Iwona Rudkowska, Mélanie Verreault, Marie Claude Vohl, Olivier Barbier

**Affiliations:** ^1^Laboratory of Molecular Pharmacology, CHU de Québec Research Centre and Faculty of Pharmacy, Laval University, Québec, QC, Canada G1V 4G2; ^2^Endocrinology and Nephrology, CHU de Québec Research Center, Québec, QC, Canada G1V 4G2; ^3^Institute of Nutraceuticals and Functional Foods (INAF), Laval University, Québec, QC, Canada G1V 0A6

## Abstract

Hypolipidemic fibrates activate the peroxisome proliferator-activated receptor (PPAR) *α* to modulate lipid oxidation and metabolism. The present study aimed at evaluating how 3 PPAR*α* agonists, namely, fenofibrate, gemfibrozil, and Wy14,643, affect bilirubin synthesis and metabolism. Human umbilical vein epithelial cells (HUVEC) and coronary artery smooth muscle cells (CASMC) were cultured in the absence or presence of the 3 activators, and mRNA, protein, and/or activity levels of the bilirubin synthesizing heme oxygenase- (HO-) 1 and biliverdin reductase (BVR) enzymes were determined. Human hepatocytes (HH) and HepG2 cells sustained similar treatments, except that the expression of the bilirubin conjugating UDP-glucuronosyltransferase (UGT) 1A1 enzyme and multidrug resistance-associated protein (MRP) 2 transporter was analyzed. In HUVECs, gemfibrozil, fenofibrate, and Wy14,643 upregulated HO-1 mRNA expression without affecting BVR. Wy14,643 and fenofibrate also caused HO-1 protein accumulation, while gemfibrozil and fenofibrate favored the secretion of bilirubin in cell media. Similar positive regulations were also observed with the 3 PPAR*α* ligands in CASMCs where HO-1 mRNA and protein levels were increased. In HH and HepG2 cells, both UGT1A1 and MRP2 transcripts were also accumulating. These observations indicate that PPAR*α* ligands activate bilirubin synthesis in vascular cells and metabolism in liver cells. The clinical implications of these regulatory events are discussed.

## 1. Introduction

Bilirubin is an endogenous bile pigment produced from heme degradation by the sequential action of the heme oxygenase (HO) and biliverdin reductase (BVR) enzymes. In humans, 2 active isoforms of heme oxygenase, namely, HO-1 and HO-2, convert heme into carbon monoxide, free iron, and biliverdin. This reaction is considered as the rate-limiting step in heme to bilirubin catabolic process [1]. BVR subsequently reduces biliverdin into bilirubin. While HO-2 is constitutively expressed, HO-1 is encoded by a highly inducible gene activated by a vast variety of endogenous and exogenous stimuli [2]. Actually, HO-1 induction is considered as a major component of the cellular response to oxidative stress, particularly in the vasculature [2]. In humans, HO-1 deficiency is related to many dangerous side effects, including injury of vascular endothelium and cardiovascular diseases [2]. Genetic polymorphisms causing low HO-1 protein expression are positively associated with increased risk for coronary events [3, 4].

Following synthesis, bilirubin binds albumin into the blood to reach the liver, where it sustains additional catabolic reactions before its elimination into the bile. The UDP-glucuronosyltransferase (UGT) 1A1 enzyme conjugates bilirubin into hydrophilic mono- and diglucuronide derivatives, which are excreted into the bile through the canalicular multidrug resistance-associated transporter (MRP) 2 protein. MRP2 is a member of the ATP-binding cassette (ABC) transporters family and is essential for bilirubin-glucuronide secretion into bile [5]. Genetic defects in the human* UGT1A1* gene are associated with unconjugated hyperbilirubinemia, which can be either asymptomatic as in individuals with Gilbert syndrome [6] or severe as in the case of Crigler-Najjar syndrome types I and II [7], depending on the remaining UGT1A1 activity. The moderate bilirubin elevation observed in Gilbert's syndrome both lowers the risk of developing coronary heart diseases [8] and accelerates the development of neonatal jaundice during the 2 first days of life [9]. Similarly, functional mutations within the* MRP2* gene result in conjugated nonhaemolytic hyperbilirubinemia, also called Dubin-Johnson syndrome [10].

Under normal circumstances, circulating levels of total, direct (i.e., conjugated), and indirect (unconjugated) bilirubin are, respectively, <17, 2–5, and 3–12 *μ*M [11]. While moderate increases (17 to 20 *μ*M) are associated to a reduction of cardiovascular events risk, bilirubin is a neurotoxic molecule at high concentration [12]. Bilirubin's atheroprotective properties relate to bilirubin's ability to efficiently scavenge reactive oxygen species (ROS), and by so to reduce low-density lipoproteins (LDL) oxidation in the vasculature [13]. On the other hand, bilirubin-induced neurotoxicity is mainly observed in newborns, where brain accumulation of unconjugated bilirubin provokes neuronal cell death and causes permanent neurologic sequel (a situation called bilirubin-induced neurologic dysfunction, BIND) (reviewed in [14]). The dual role of bilirubin as an atheroprotective agent and neurotoxic molecule renders essential a tight control of its metabolism.

The peroxisome proliferator-activated receptor (PPAR) *α* belongs to the PPAR family of lipid sensors. With 2 other members, PPAR*δ* and *γ*, these ligand-activated transcription factors regulate lipid and fatty acid homeostasis, as well as energy storage and expenditure [15]. Upon ligand activation, these receptors form an active heterodimer with their partner retinoic X receptor (RXR) and bind to the promoter regions of target genes on specific DNA sequences called PPAR response elements (PPRE) [16]. PPAR*α* target genes play key roles in lipid transport, fatty acid *β*-oxidation, lipogenesis, lipoprotein uptake, and metabolism, as well as in cholesterol transport and elimination [17]. PPAR*α* is mainly expressed in the liver and heart, where it is activated by endogenous activators such as fatty acid derivatives (i.e., eicosanoids, palmitic, oleic, and linoleic acids) or exogenous ligands, such as the Wy14,643 compounds or fibrate drugs (i.e., gemfibrozil, clofibrate, ciprofibrate, and fenofibrate) [18]. These fibric acid derivatives have been used in clinics since the mid-1960s to lower plasma triglyceride (TG) levels in patients with atherogenic dyslipidemia [19].

Several investigations identified PPAR*α* as an important modulator for genes controlling bilirubin synthesis (HO-1) and metabolism (UGT1A1 and MRP2) [20–22], leading to the hypothesis that fibrates coordinately control the synthesis and metabolism of this bile pigment. However, all these studies were performed in different experimental settings, using variable ligands, doses, experimental models, and analytical tools. Considering the agonist- and/or cell type-dependent manner in which PPAR*α* agonists regulate their target genes [23, 24], we comprehensively and comparatively analyzed the ability of gemfibrozil, fenofibrate, and Wy14,643 compound to regulate HO-1, BVR, UGT1A1, and/or MRP2 expression in relevant hepatic and vascular cell models.

## 2. Materials and Methods

### 2.1. Materials

Wy14,643 (pirinixic acid, 4-Chloro-6-(2,3-xylidino)-2-pyrimidinylthioacetic acid) and fenofibrate were from Sigma (St. Louis, MO) and ICN Pharmaceuticals, Inc. (Montréal, Canada), respectively. Gemfibrozil was from Pfizer Canada (Kirkland, Canada). Fetal bovine serum (FBS) and other cell culture reagents were from Invitrogen (Burlington, Canada). The SYBR Green PCR mix was purchased from Applied Biosystems (Foster City, CA). Protein assay reagents were obtained from Bio-Rad Laboratories Inc. (Marnes-la-Coquette, France). The anti-HO-1 antibody was from Santa Cruz (Santa Cruz, CA). The anti-actin antibody was purchased from Sigma and the anti-rabbit IgG antibody was from GE Healthcare (Piscataway Township, NJ).

### 2.2. Cell Culture

Human umbilical vein endothelial cells (HUVEC) and coronary artery smooth muscle cells (CASMC) were from Lonza (Walkersville, MD). HUVECs were cultured in the endothelial cell growth medium (EGM) according to the manufacturer's instructions (Lonza). For treatment with DMSO (vehicle; 0.1%, v/v), gemfibrozil (250 *μ*M), fenofibrate (250 *μ*M), and Wy14,643 (75 *μ*M) cells were platted in 12-well plates (1.2 × 10^5^ cells/well) for mRNA determination, in 6-well plates (2.5 × 10^5^ cells/well) for bilirubin secretion measurement, or in 10 cm petri dishes (6.0 × 10^5^ cells/dish) for Western blot analyses. Treatments were performed in EGM-0.2% FBS for the indicated duration. CASMCs were cultured in the smooth muscle growth medium-2 (SmGM-2) according to the manufacturer's instructions (Lonza) and platted in 6-well plates (1.25 × 10^5^ cells/well) or 10 cm-petri dishes (4.0 × 10^5^ cells/dish). Cells were treated with DMSO (vehicle, 0.1%, v/v), 250 *μ*M fenofibrate, 250 *μ*M gemfibrozil, or 75 *μ*M Wy14,643 for up to 48 H. Human hepatoma HepG2 cells were from the American Type Culture Collection (Rockville, MD) and cryopreserved human hepatocytes (HH) from 2 individual donors were from Celsis: In Vitro Technologies (Baltimore, MD). Donor 1 was an African-American man at the age of 46 (cause of death: anoxia), while donor 2 was a Caucasian woman at the age of 40 (cause of death: drug overdose). HepG2 cells were grown as described [26]. Cells (3 × 10^5^ per well) were plated in 12-well plates and treated with DMSO (vehicle; 0.1%, v/v), 250 *μ*M fenofibrate, 250 *μ*M gemfibrozil, or 75 *μ*M Wy14,643 for up to 48 H. Human hepatocytes in primary culture were plated in 24-well plates (3.5 × 10^5^ cells/well) and maintained in InVitroGro CP medium for 48 H with medium change after 24 H as recommended by the supplier (Celsis: In Vitro Technologies). Afterwards, cells were treated with DMSO (vehicle; 0.1%, v/v), gemfibrozil (200 *μ*M), fenofibrate (250 *μ*M), or Wy14,643 (75 *μ*M) in the InVitroGro HI medium (Celsis: In Vitro Technologies) for 24 H.

### 2.3. Messenger RNA Levels Determination

Total RNA was isolated from control or treated cells according to the TRI Reagent acid: phenol protocol as specified by the supplier (Molecular Research Center Inc., Cincinnati, OH). The reverse transcription reaction was performed using 200 units of Superscript II (Invitrogen, Burlington, Canada) with up to 1 *μ*g of total RNA and 7.5 ng of random hexamere (Roche, Laval, Canada) at 42°C for 50 min, as described in [26]. The real-time PCR reactions were performed using an ABI Prism 7500 instrument from Applied Biosystems (Foster City, CA). For each reaction, the final volume of 20 *μ*L was comprised of 10 *μ*L of SYBR Green PCR Mix, 2 *μ*L of each primer (Table 1), and 6 *μ*L of the 1/500 dilution of RT products. Conditions for real-time PCR were 95°C for 20 sec, 95°C for 30 sec, and annealing temperature for 20 sec for 40 cycles. The specific amplification was ensured by direct sequencing of PCR products. Threshold cycle (Ct) values were analyzed using the comparative Ct (ΔΔCt) method as recommended by the manufacturer (Applied Biosystems). The amount of target gene (2^−ΔΔCt^) was obtained by normalizing to the endogenous reference 28S and was expressed relatively to vehicle-treated cells set at 1. For each gene, the amplification efficiency and the accuracies of ΔΔCt of target genes compared with 28S were tested using 2 to 5 log of concentrations of cDNA produced from cell purified mRNA.

### 2.4. Western Blot Analysis

Control and treated cells were washed in ice-cold PBS and harvested in ice-cold PBS containing 0.5 mM dithiothreitol. Total proteins (20–25 *μ*g) were size-separated on 10% SDS-polyacrylamide gels and immunoblotted with an anti-HO-1 antibody (1 : 400). An anti-rabbit IgG donkey antibody (1 : 10,000) conjugated with peroxidase was used as the second antibody. Immunocomplexes were visualized on hyperfilm. The same membranes were then rehybridized with an anti-actin (1 : 2,000) antibody as a loading control assessment.

### 2.5. Bilirubin Determination in Culture Media

Culture media were analyzed for bilirubin through liquid chromatography coupled to tandem mass spectrometry (LC-MS/MS). Briefly, 500 *μ*L media were added to a mixture composed of 100 *μ*L butylated hydroxytoluene (BHT, 1% w/v methanol), 100 *μ*L ascorbic acid (0.4% w/v methanol), 500 *μ*L H_2_O, 25 *μ*L HCl (0.2 M), and 100 *μ*L mesobilirubin (100 ng/L, internal standard, Sigma-Aldrich Inc.). After liquid : liquid extraction with 2 mL of chloroform, the organic phase was evaporated under nitrogen, and analytes were dissolved in 200 *μ*L of a methanol : chloroform : water (81 : 10 : 9) solution. The chromatographic separation was achieved with an Alliance 2690 LC apparatus (Waters, Milford, MA) equipped with an ACE-3 C18 HL 100 × 4.6 mm (3 *μ*m particles) column, using a 5 mM ammonium formate methanol gradient. Bilirubin and mesobilirubin detection was performed through tandem mass spectrometry with an API4000 instrument (Applied Biosystems-Sciex) mass spectrometer. The limit of quantification (LOQ) was 1 ng/mL. Bilirubin concentration values were calculated as the ratio of areas under curve for bilirubin versus mesobilirubin.

### 2.6. Statistical Analyses

All data are presented as mean ± standard deviation (SD). Comparisons between two groups were performed using the two-tailed Student's *t*-test with the JMP V4.0.2 software (SAS Institute, Cary, NC).

## 3. Results

### 3.1. Gemfibrozil Stimulates HO-1, UGT1A1, and MRP2 mRNA Expression

**Figure 1:**
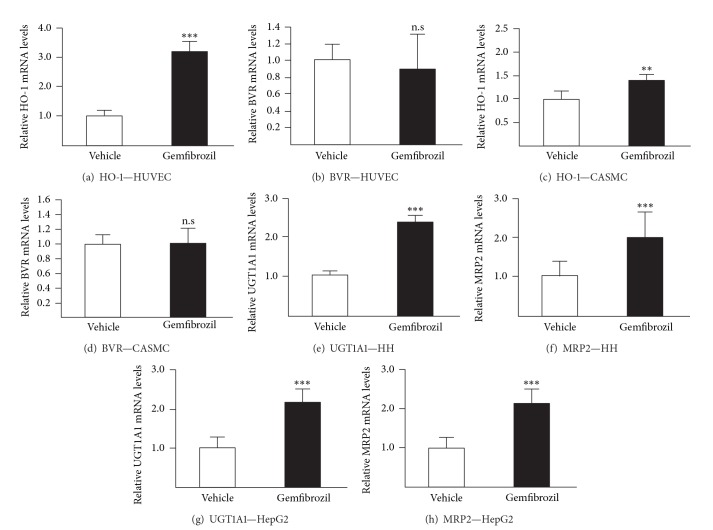
Gemfibrozil increases HO-1, UGT1A1, and MRP2 mRNA levels in human vascular and hepatic cell models. Human umbilical vein endothelial cells (HUVEC) (a and b), coronary artery smooth muscle cells (CASMC) (c and d), and HepG2 cells (g and h) were treated with vehicle (DMSO, 0.1%, v/v) or gemfibrozil (250 *μ*M) for 24 H. Human hepatocytes (HH) from donor 1 (e and f) were treated with vehicle (DMSO, 0.1%, v/v) or gemfibrozil (200 *μ*M) for 24 H. Transcript levels were quantified from total RNA through quantitative RT-PCR analyses and normalized with the housekeeping RNA 28S. Values (mean ± SD) are expressed relatively to control (vehicle) set at 1. Statistically significant differences between vehicle- and gemfibrozil-treated cells are indicated by asterisks (Student's *t*-test: **P < 0.01; ***P < 0.001; n.s: nonsignificant).

We first tested whether gemfibrozil affects mRNA expression of the bilirubin synthesizing HO-1 and BVR enzymes in vascular endothelial cells. After 24 H, the drug caused a 3-time (*P* < 0.001) accumulation of HO-1 mRNA levels (Figure 1(a)). Similar inductions were also observed in CASMCs (Figure 1(c); 1.4-fold; *P* < 0.01). By contrast, expression of the biliverdin to bilirubin converting BVR enzyme was not affected in both cell models (Figures 1(b) and 1(d)). We next investigated whether UGT1A1 and MRP2 expression was also responding to gemfibrozil exposure in 2 human liver cell lines. In both human hepatocytes and hepatoma HepG2 cells, a 24 H treatment with gemfibrozil resulted in 2-fold increases in UGT1A1 (Figures 1(e) and 1(g), *P* < 0.001) and MRP2 mRNA levels (Figures 1(f) and 1(h); *P* < 0.001). These results indicate that gemfibrozil positively affects the expression of the bilirubin synthesising HO-1 enzyme in vascular cells and stimulates the metabolizing (UGT1A1) and elimination systems (MRP2) in liver cells.

### 3.2. Fenofibrate and Wy14,643 also Activate Genes Controlling Bilirubin Synthesis and Metabolism

**Figure 2:**
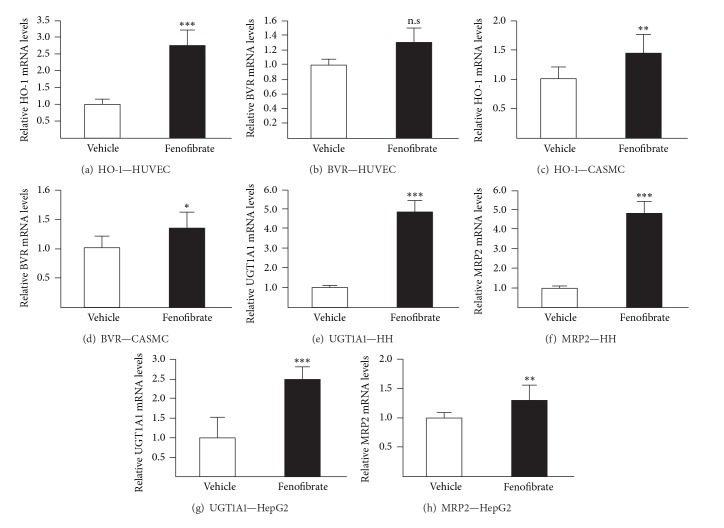
Fenofibrate increases HO-1, UGT1A1, and MRP2 mRNA levels in human vascular and hepatic cell models. Human umbilical vein endothelial cells (HUVEC) (a and b), coronary artery smooth muscle cells (CASMC) (c and d), human hepatocytes (HH; donor 2) (e and f), and HepG2 cells (g and h) were treated with vehicle (DMSO, 0.1%, v/v) or fenofibrate (250 *μ*M) for 24 H (HUVEC and HH) or 48 H (CASMC and HepG2). Messenger RNA levels were quantified from total RNA through quantitative RT-PCR analyses and normalized with the housekeeping RNA 28S. Values (mean ± SD) are expressed relatively to control (vehicle) set at 1. Statistically significant differences between vehicle- and fenofibrate-treated cells are indicated by asterisks (Student's *t*-test: *P < 0.05; **P < 0.01; ***P < 0.001; n.s: nonsignificant).

**Figure 3:**
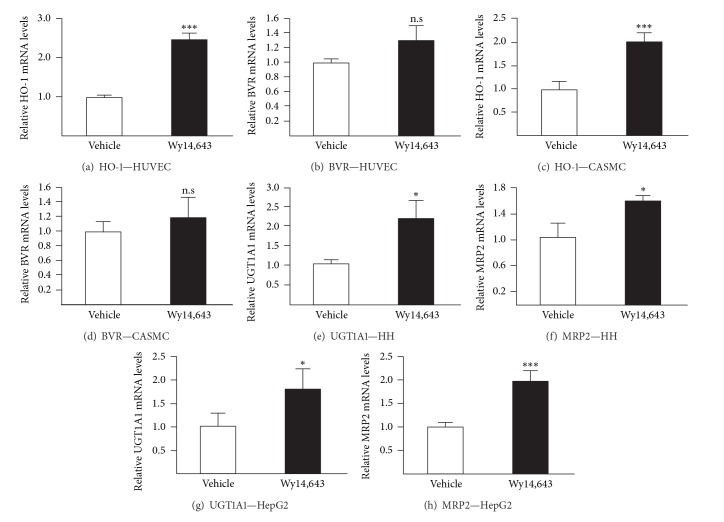
The high affinity PPAR*α* agonist Wy14,643 increases HO-1, UGT1A1, and MRP2 mRNA levels in human vascular and hepatic cell models. Human umbilical vein endothelial cells (HUVEC) (a and b), coronary artery smooth muscle cells (CASMC) (c and d), human hepatocytes (HH; donor 1) (e and f), and HepG2 cells (g and h) were treated with vehicle (DMSO, 0.1%, v/v) or Wy14,643 (75 *μ*M) for 24 H. HO-1, BVR, UGT1A1, and MRP2 mRNA levels were quantified from total RNA through quantitative RT-PCR analyses and normalized with the housekeeping RNA 28S. Values (mean ± SD) are expressed relatively to control (vehicle) set at 1. Statistically significant differences between vehicle- and Wy14,643-treated cells are indicated by asterisks (Student's *t*-test: *P < 0.05; ***P < 0.001; n.s: nonsignificant).

To further confirm the role of PPAR*α* in the gemfibrozil-dependent induction of HO-1, UGT1A1, and MRP2 mRNA levels, we next investigated whether fenofibrate and Wy14,643 exert any effects on their expression. Both molecules caused a significant accumulation of HO-1 transcripts in HUVECs (Figures 2(a) and 3(a)) and CASMCs (Figures 2(b) and 3(b)). As with gemfibrozil, BVR mRNA levels remained unchanged in cells exposed to Wy14,643 (Figures 3(b) and 3(d)). Interestingly, fenofibrate caused significant accumulation of this transcript (*P* < 0.01) but only in CASMCs (Figure 2(d)).

In liver cells, fenofibrate and Wy14,643 also acted as positive modulators of UGT1A1 and MRP2 genes expression (Figures 2(e), 2(f), 2(g), 2(h), 3(e), 3(f), 3(g), and 3(h)). The strongest inductions were observed in fenofibrate-treated hepatocytes where both UGT1A1 (*P* < 0.001) and MRP2 (*P* < 0.001) mRNAs sustained 5-fold accumulations (Figures 2(e) and 2(f)). The lowest response corresponded to the 1.3-fold MRP2 mRNA accumulation in fenofibrate-treated HepG2 cells (Figure 2(h), *P* < 0.01).

Overall, these results indicate that both clinically relevant (gemfibrozil, fenofibrate) and high affinity (Wy14,643) PPAR*α* ligands act as inducers of genes controlling bilirubin synthesis and metabolism.

### 3.3. PPAR*α* Ligands Differentially Affect HO-1 Protein Levels and/or Bilirubin Secretion in Vascular Cells

**Figure 4:**
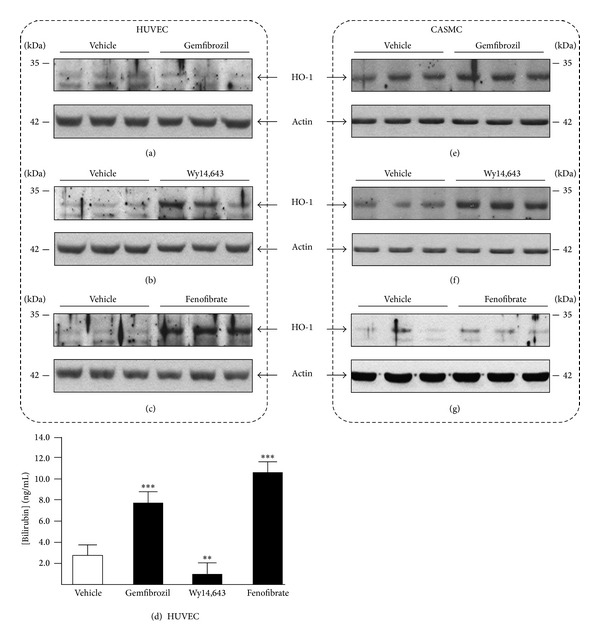
PPAR*α* ligands differentially affect HO-1 protein levels and/or bilirubin secretion in human umbilical vein endothelial cells (HUVEC) and coronary artery smooth muscle cells (CASMC). HUVECs (a–d) and CASMCs (e–g) were cultured in the presence of vehicle (DMSO, 0.1%, v/v), 250 *μ*M gemfibrozil (a, d, and e), 75 *μ*M Wy14,643 (b, d, and f), or 250 *μ*M fenofibrate (c, d, and g) for 48 H. ((a–c) and (e–g)) HO-1 protein levels were visualized in cell homogenates (20 *μ*g–25 *μ*g) through immunoblotting using the anti-HO-1 (1 : 400 dilution) antibody. The same membranes were subsequently hybridized with an anti-actin antibody (1 : 2,000 dilution) to ensure the equal loading of each lane. (d) Bilirubin concentration was determined in culture media through liquid chromatography coupled to tandem mass spectrometry. Statistically significant differences are indicated by asterisks (Student's *t*-test: **P < 0.01; ***P < 0.001).

To further evaluate the consequences of the fibrate-dependent activation of HO-1 mRNA expression, HUVECs were subsequently investigated for HO-1 protein levels and bilirubin secretion in culture media (Figure 4). While HUVECs exposed to 250 *μ*M gemfibrozil for 48 H displayed no major changes in HO-1 protein contents (Figure 4(a)), homogenates from cells cultured for the same duration but in the presence of Wy14,643 (75 *μ*M) or fenofibrate (250 *μ*M) displayed convincing accumulation of the heme oxygenase protein when compared to samples from control cells (vehicle) (Figures 4(b) and 4(c)). Interestingly, an agonist-specific pattern was also observed when analyzing bilirubin secretion (Figure 4(d)). While gemfibrozil and fenofibrate led to 2.3- and 3.0-fold increases in culture media bilirubin concentration, Wy14,643 had the opposite effect since the bile pigment was actually 1.6-fold less abundant than in media from control cells.

To evaluate whether such discrepancies could reflect an inadequacy of the cell model, similar experiments were then performed with CASMCs (Figures 4(e)–4(g)). In these cells, all 3 PPAR*α* ligands caused HO-1 proteins accumulation. Interestingly, while similar amounts of cell homogenates and antibody were used for Western blotting, detection of the HO-1 protein in HUVEC homogenates required a longer film exposure than those from CASMCs, suggesting that, in baseline, HO-1 is more abundant in the smooth muscle model than in the endothelial ones. However, the opposite was observed when measuring bilirubin formation since this molecule was only detected at concentrations below the limit of quantification in media from untreated CASMCs (data not shown). Interestingly, exposing these cells to gemfibrozil, Wy14,643 and fenofibrate resulted in an unquantifiable but convincing increase of the bilirubin's AUC in LC-MS/MS analyses (data not shown).

In summary, even when considering technical limitations, these observations support the idea that PPAR*α* ligands are positive regulators of HO-1 expression and activity in HUVECs and CASMCs, when taken together.

## 4. Discussion

**Figure 5:**
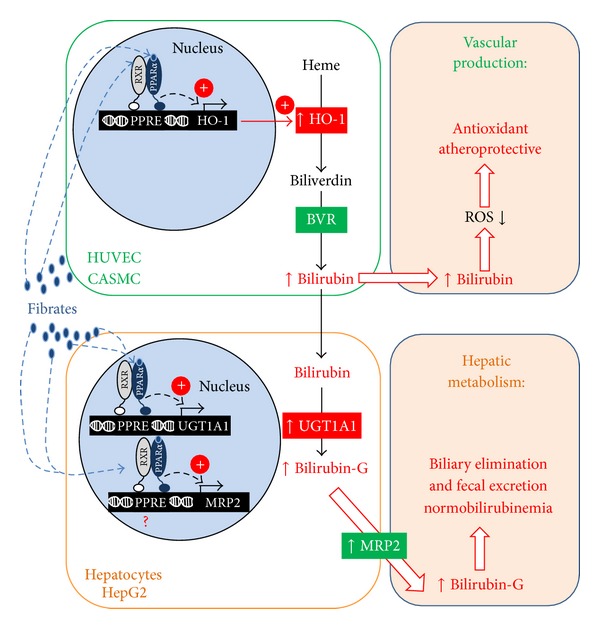
Coordinated regulation of bilirubin synthesis and metabolism by PPAR*α* agonists has antiatherosclerotic effects in the vasculature and normobilirubinemic consequences in the liver. Results presented here suggest that PPAR*α* agonists, such as fenofibrate, gemfibrozil, and Wy14,643, activate the synthesis of bilirubin in the vasculature and its elimination in the liver. In vascular cells (HUVEC and CASMC), fibrates activate PPAR*α*, which in turn binds to PPREs located in the promoter region of the* HO-1* gene [20]. This leads to an increased HO-1 expression and heme-to-bilirubin conversion. As a potent antioxidant, bilirubin scavenges reactive oxygen species, and by so exerts atheroprotective effects. In liver cells (hepatocytes), fibrates also activate PPAR*α*, which in turn binds to PPREs located in the promoter region of the* UGT1A1* [25] and potentially* MRP2* genes (this remains to be established). These regulatory events lead to increased bilirubin-glucuronide production and biliary secretion and by so contribute to reduce systemic accumulation of bilirubin. BVR: biliverdin reductase; CASMC: coronary artery smooth muscle cells; G: glucuronide; HO-1: heme oxygenase-1; HUVEC: human umbilical vein endothelial cells; MRP2: multidrug resistance-associated transporter protein 2; PPAR: peroxisome proliferator-activated receptor; PPRE: PPAR response element; ROS: reactive oxygen species; and UGT1A1: UDP-glucuronosyltransferase 1A1.

The present investigations provide a comprehensive analysis of PPAR*α* agonists' impacts on bilirubin synthesis and metabolism. Our observations indicate that each tested PPAR*α* activator regulates genes controlling bilirubin synthesis in vascular cells and its metabolism in hepatic cells. Considering the antioxidant properties of bilirubin, it can be envisioned that this coordination allows beneficial local antiatherosclerotic effects, while avoiding toxic accumulation of the bile pigment in the systemic circulation (Figure 5).

We observed that the 3 PPAR*α* agonists assayed with HUVECs and/or CASMCs were able to upregulate HO-1 mRNA expression, protein content, and/or activity levels. Similar inductions were previously reported with fenofibrate and Wy14,643 in HUVEC and human vascular smooth muscle cells (VSMCs) [20]. In addition, fenofibrate also prevents the reduction of HO-1 transcript levels caused by exposure of human pulmonary artery endothelial cells to serum from patients with stable advanced chronic heart failure [27]. However, to the best of our knowledge, the present study provides the first experimental evidences that gemfibrozil also positively regulates HO-1 expression in cell models of the human vasculature. Thus, our investigations further support the idea that PPAR*α* agonists act as positive regulators of the human* HO-1* gene in cells from the vascular wall. These regulatory events are thought to participate to the therapeutic benefits observed with fibrate drugs in the context of several pathological situations. Indeed, a number of experimental evidences demonstrate the contribution of the HO-1 induction to the positive effects exerted by PPAR*α* agonists against: (i) the hepatotoxicity caused by iron deposition in the liver [28]; (ii) renal injury caused by ischemia/reperfusion [29]; (iii) iron-induced cardiomyopathy [30]; and (iv) carboplatin-induced nephrotoxicity [31]. Thus, it is tempting to speculate that in vascular wall cells, HO-1 induction also participates to the antiatherosclerotic effects of fibrate drugs. This idea is supported by the recent observation that HO-1 inhibitors reduce the anti-inflammatory and antiproliferative effects of PPAR*α* activators in human VSMCs [20]. Actually, the atheroprotective role played by HO-1 has been deeply documented through gene transfer experiments involving a series of relevant animal models [32–34]. These studies established that HO-1 overexpression reduces the vascular inflammatory response in rat VSMCs [33] and attenuates atherosclerosis development in apoE deficient mice [34] and the rat aortic transplant model [32]. Thus, the PPAR*α*-dependent induction of HO-1 in vascular wall cell models may also generate antiatherosclerotic effects (Figure 5).

In addition to the bile pigment, the heme-to-bilirubin conversion system also generates carbon monoxide and free iron [35]. The relative contribution of each of these end-products in HO-1 mediated atheroprotection still remains to be clarified [35]. However, an impressive number of clinical studies illustrate the inverse relationship linking serum total bilirubin concentration to the cardiovascular risk, at least in stable coronary conditions (reviewed in [36]). To quote only a few recent examples, serum bilirubin is inversely correlated with: (i) the severity of disease in patients with stable coronary artery disease [37]; (ii) the carotid artery intima/media thickness in nondiabetic and type 2 diabetic subjects [38]; and (iii) the clinical outcomes at the time of the 5-year follow-up of patients with cardiac syndrome X [39]. The protective properties of bilirubin relate to its powerful antioxidant capabilities, and its efficiency at scavenging peroxyl radicals [40]. This characteristic is of particular interest in the vasculature where bilirubin prevents a precursor event in atherogenesis, namely, the oxidation of LDL particles [40]. Accordingly, high serum bilirubin levels are associated with reduced circulating levels of oxidized LDL in healthy and Gilbert syndrome individuals [41, 42]. Thus, in patients with stable coronary conditions, high bilirubin levels are associated with reduced oxLDL formation and favorable endothelial function [43]. Here, we observe that both clinically relevant fibrates (gemfibrozil and fenofibrate) caused a significant increase in bilirubin production in culture media of vascular endothelial cells. Such an accumulation of unconjugated bilirubin (UCB) in media has been previously associated with intracellular content of UCB in a linear manner and diminution of lipid peroxidation susceptibility in UCB-treated rat brains [44]. It can therefore be postulated that fibrate treatment increases bilirubin production in the vascular wall and by so contribute to reduce LDL oxidation and atherosclerotic plaque formation (Figure 5). One can argue that such a mechanism could be minimized by the low BVR modulation observed in PPAR*α* agonists treated cells. However, it is well established that HO-1 catalyzes the rate-limiting process in heme degradation (reviewed in [1]). Actually, the absence of BVR modulation in our experiments is consistent with the fact that BVR is a noninducible protein [45], which mainly sustains posttranslational regulatory processes, such as autophosphorylation, instead of transcriptional gene controls [45].

Beyond HO-1 expression in vascular wall cells, our investigations also demonstrate that gemfibrozil, fenofibrate, and Wy14,643 positively regulate UGT1A1 and MRP2 expression in human cell models. In humans, UGT1A1 is the unique glucuronosyltransferase enzyme catalyzing the conversion of bilirubin into highly hydrophilic and easily excretable glucuronide derivatives [7], and MRP2 ensures the export of these derivatives into the bile (Figure 5) [5]. The present observations therefore indicate that PPAR*α* activators coordinately activate the 2-step bilirubin detoxification system in liver cells. These observations are fully consistent with previous investigations revealing that gemfibrozil [23], fenofibrate [23], and Wy14,643 [25] cause significant accumulations of UGT1A1 transcripts in hepatocytes. By contrast, the PPAR*α*-dependent regulation of MRP2 expression sustained some controversy in the recent years. Although bezafibrate exposure caused significant increase in MRP2 mRNA in human hepatic HepaRG cells [22] as well as in liver cells from treated mice [46], it was unclear whether such effects were PPAR*α*-dependent [22]. Other* in vivo* investigations using ciprofibrate also revealed conflicting results. While Kok and colleagues [47] and Aleksunes and Klaassen [48] reported PPAR*α*-dependent induction of Mrp2 mRNA expression in livers from ciprofibrate-treated mice, other studies involving clofibric acid and ciprofibrate revealed nonsignificant reduction of the Mrp2 proteins in rat liver [49] and mRNA in mice liver [50], respectively. In summary, results of the current study confirm the positive effects that PPAR*α* agonists exert on UGT1A1 expression and support a similar response of the* MRP2* gene, at least in human hepatocytes and hepatoma HepG2 cells.

As for HO-1, the PPAR*α*-dependent induction of UGT1A1 expression is pharmacologically relevant for the treatment of hyperbilirubinemia. Indeed, the improved glucuronidation activity resulting from these regulatory events favors bilirubin elimination [51]. Unconjugated hyperbilirubinemia (i.e., jaundice) is a common clinical problem during the neonatal period which may result in brain damage, even in healthy full-term newborns [52]. While the main treatment for hyperbilirubinemic neonates remains phototherapy [53], the UGT1A1 inducing effects of fibrate drugs encouraged their evaluation for bilirubin elimination. These investigations suggested that clofibrate is efficient in reducing both total serum bilirubin and phototherapy duration (reviewed in [54]). However, such a therapeutic benefit appears to be fibrate-dependent since, in similar studies performed with preterm and term neonates with moderate jaundice, gemfibrozil failed to reduce the phototherapy duration or peak bilirubin levels [55]. Furthermore, as highlighted in a recent Cochrane evaluation [54], most of clinical studies performed with clofibrate were conducted in Iran, and the benefits of the therapy remain to be ascertained through larger trials performed in different countries.

## 5. Conclusion

In conclusion, the present study demonstrates that PPAR*α* activators, such as gemfibrozil, fenofibrate, and Wy14,643, coordinately stimulate the gene expression of HO-1 in cell models of the human vasculature and of UGT1A1 and MRP2 in human liver cells. These observations support the idea that, by modulating both bilirubin synthesis and catabolism, fibrate drugs exert antiatherosclerotic effects in the vasculature and antihyperbilirubinemic properties in the liver (Figure 5).

## Figures and Tables

**Table 1:** Primers and conditions used for real-time RT-PCR experiments.

Gene	Primers	Concentration (*µ*M)	Annealing temperature (°C)
HO-1	Sense: 5′-AAGATTGCCCAGAAAGCCCTGGAC	2.50	65°C
	Antisense: 5′-AACTGTCGCCACCAGAAAGCTGAG		
BVR	Sense: 5′-TTGGCGTGGTGGTGGTTGGTGTT	2.00	63°C
	Antisense: 5′-CTCCACCTCTTGGCTGGAAAGAG		
UGT1A1	Sense: 5′-GAGAGAGGTGACTGTCCAGGAC	1.25	63°C
	Antisense: 5′-CAAATTCCTGGGATAGTGGATTTT		
MRP2	Sense: 5′-CAAACTCTATCTTGCTAAGCAGG	2.00	59°C
	Antisense: 5′-TGAGTACAAGGGCCAGCTCTA		
28S	Sense: 5′-AAACTCTGGTGGAGGTCCGT	2.00	60°C
	Antisense: 5′-CTTACCAAAAGTGGCCCACTA		
